# Supplementary material for: Defoliation-induced changes in foliage quality may trigger broad-scale insect outbreaks
Source: Commun Biol. 2022 May 16;5:463. doi: 10.1038/s42003-022-03407-8 (PMC9110339; doi:10.1038/s42003-022-03407-8)
Supplement: Supplementary file 2 — Supplementary Information [file 42003_2022_3407_MOESM2_ESM.pdf]

Supplementary Materials

Defoliation-induced changes in foliage quality trigger broad-scale insect outbreaks

Authors
Louis De Grandpré*, Maryse Marchand, Daniel D. Kneeshaw, David Paré, Dominique Boucher, Stéphane Bourassa, David Gervais, Martin Simard, Jacob M. Griffin and Deepa S. Pureswaran

*Corresponding author. Email: louis.degrandpre@canada.ca

This PDF file includes:

Supplementary Tables 1 to 8

Supplementary Table 1 Estimated marginal means of annual defoliation (%), larval density (L4 kg^{-1} of branches), annual litterfall ($kg\ ha^{-1}\ yr^{-1}$) and cumulative tree mortality ($m^2\ ha^{-1}$) estimated from linear least-squares regression models (n = 10) and pairwise comparisons among stand composition types. Values in parentheses indicate the 95% confidence interval adjusted for simultaneous inference and stars indicate estimates significantly different from zero at $\alpha = 0.05$.

	Stand type	DF	Defoliation	Larval density	Litterfall	Mortality
**Estimated marginal means**	Bf-stand[*]	7	50 (38, 63)*	144 (112, 176)*	1410 (1018, 1802)*	17.2 (10.9, 23.5)*
	Mixed[‡]	7	45 (33, 58)*	131 (100, 163)*	1124 (732, 1517)*	16.3 (10.0, 22.6)*
	Bs-stand[†]	7	31 (20, 42)*	67 (39, 95)*	879 (539, 1218)*	5.0 (-0.4, 10.5)
**Pairwise comparisons in means**	Bf vs mixed	7	5 (-12, 22)	12 (-31, 56)	286 (-250, 822)	0.9 (-7.7, 9.5)
	Bf vs Bs	7	19 (3, 35)*	77 (36, 118)*	531 (30, 1033)*	12.2 (4.2, 20.2)*
	Mixed vs Bs	7	14 (-2, 30)	65 (24, 105)*	246 (-256, 747)	11.3 (3.3, 19.3)*
**Adjusted R^2**			0.58	0.80	0.46	0.73
**F statistic (df = 2,7)**			7.16*	18.73*	4.88*	13.06*

[*]Bf: Balsam fir-dominated stands, where basal area ($m^2\ ha^{-1}$) of balsam fir represents > 75% of total coniferous tree basal area in the stand. [†]Bs: Black spruce-dominated stands, where basal area ($m^2\ ha^{-1}$) of black spruce represents > 75% of total coniferous tree basal area in the stand. [‡]Mixed stands, where balsam fir and black spruce each represent between 25% and 75% of total coniferous basal area.

Supplementary Table 2 Slopes of the relationship between nutrient concentrations (nitrogen, phosphorus and potassium, in g kg^{-1}) in litterfall and outbreak progression (time, in years) for each stand type and litter type, estimated from the linear mixed-effects models (n = 1360), and pairwise slope comparisons among stand types. For P and K, slope comparisons are the same across all litter types as the three-way interaction between stand type, litter type and time was not significant and therefore removed from the models. Values in parentheses indicate the 95% confidence interval adjusted for simultaneous inference and stars indicate estimates significantly different from zero at $\alpha = 0.05$.

	**Stand type**	**Litter type**	**n**	**Nitrogen**	**Phosphorus**	**Potassium**
Slope estimates (year)	Bf-stand	Needles	147	0.8 (0.7, 1.0)*	0.07 (0.06, 0.08)*	0.0001 (-0.01, 0.01)
	Mixed	Needles	147	0.7 (0.5, 0.8)*	0.06 (0.05, 0.07)*	-0.01 (-0.02, -0.0002)*
	Bs-stand	Needles	196	0.4 (0.3, 0.5)*	0.04 (0.03, 0.05)*	-0.03 (-0.04, -0.02)*
	Bf-stand	Leaves	138	1.0 (0.8, 1.2)*	0.07 (0.05, 0.08)*	-0.08 (-0.13, -0.04)*
	Mixed	Leaves	90	0.6 (0.4, 0.9)*	0.06 (0.05, 0.07)*	-0.10 (-0.14, -0.05)*
	Bs-stand	Leaves	152	0.1 (-0.05, 0.3)	0.04 (0.03, 0.05)*	-0.11 (-0.16, -0.07)*
	Bf-stand	Frass	147	1.0 (0.8, 1.2)*	0.1 (0.11, 0.14)*	0.02 (0.005, 0.04)*
	Mixed	Frass	147	0.7 (0.5, 0.9)*	0.11 (0.10, 0.12)*	0.01 (-0.003, 0.03)
	Bs-stand	Frass	196	0.7 (0.5, 0.8)*	0.10 (0.08, 0.11)*	-0.006 (-0.02, 0.005)
Slope comparisons among stand types (year)	Bf-stand vs mixed	Needles		0.2 (-0.1, 0.4)	0.009 (-0.005, 0.02)	0.01 (-0.006, 0.03)
		Leaves		0.4 (0.1, 0.7)*		
		Frass		0.3 (0.1, 0.6)*		
	Bf-stand vs Bs-stand	Needles		0.5 (0.3, 0.7)*	0.02 (0.01, 0.04)*	0.03 (0.01, 0.05)*
		Leaves		0.9 (0.7, 1.2)*		
		Frass		0.4 (0.1, 0.6)*		
	Mixed vs Bs-stand	Needles		0.3 (0.1, 0.5)*	0.02 (0.005, 0.03)*	0.02 (0.003, 0.03)*
		Leaves		0.5 (0.2, 0.8)*		
		Frass		0.03 (-0.2, 0.3)		

Supplementary Table 3 Slopes of the relationship between annual nutrient fluxes (nitrogen, phosphorus and potassium, in kg ha^{-1}) in litterfall and outbreak progression (time, in years) for each stand type and litter type, estimated from the linear mixed-effects models (n = 980), and pairwise slope comparisons among stand types. The three-way interaction between stand type, litter type and time was significant for all models. Values in parentheses indicate the 95% confidence interval adjusted for simultaneous inference and stars indicate estimates significantly different from zero at α = 0.05.

	Stand type	Litter type	n	Nitrogen	Phosphorus	Potassium
Slope estimates (year)	Bf-stand	Needles	147	1.4 (1.0, 1.8)*	0.10 (0.07, 0.13)*	0.02 (-0.003, 0.04)
	Mixed		147	0.6 (0.4, 0.9)*	0.05 (0.03, 0.06)*	-0.003 (-0.02, 0.01)
	Bs-stand		196	0.5 (0.4, 0.6)*	0.04 (0.03, 0.05)*	0.004 (-0.006, 0.01)
	Bf-stand	Frass	147	0.5 (0.3, 0.7)*	0.06 (0.04, 0.08)*	0.02 (0.005, 0.03)*
	Mixed		147	0.6 (0.5, 0.7)*	0.05 (0.04, 0.06)*	0.02 (0.02, 0.03)*
	Bs-stand		196	0.4 (0.3, 0.4)*	0.04 (0.04, 0.05)*	0.009 (0.004, 0.01)*
Slope comparisons among stand types(year)	Bf-stand vs mixed	Needles		0.7 (0.3, 1.2)*	0.05 (0.02, 0.09)*	0.02 (-0.004, 0.05)
	Bf-stand vs Bs-stand			0.9 (0.5, 1.3)*	0.06 (0.03, 0.09)*	0.01 (-0.009, 0.04)
	Mixed vs Bs-stand			0.2 (-0.1, 0.4)	0.004 (-0.01, 0.02)	-0.008 (-0.03, 0.01)
	Bf-stand vs mixed	Frass		-0.1 (-0.3, 0.1)	0.006 (-0.02, 0.03)	-0.005 (-0.02, 0.01)
	Bf-stand vs Bs-stand			0.2 (-0.1, 0.4)	0.01 (-0.007, 0.03)	0.008 (-0.005, 0.02)
	Mixed vs Bs-stand			0.3 (0.1, 0.4)*	0.007 (-0.006, 0.02)	0.01 (0.005, 0.02)*

Supplementary Table 4 Estimated marginal means (EMMs) of nutrient concentrations (N, P and K, in g kg^{-1}) in litterfall estimated from the linear mixed-effects models (n = 1360) and pairwise comparisons among stand types at the beginning of the study period. As litter type and stand type interact with year in all three models, these EMMs and their comparisons are valid for 2011 only. Values in parentheses indicate the 95% confidence interval adjusted for simultaneous inference and stars indicate estimates significantly different from zero at $\alpha = 0.05$.

	**Stand type**	**Year**	**Litter type**	**DF**	**Nitrogen**	**Phosphorus**	**Potassium**
Estimated marginal means (EMMs)	Bf-stand	2011	Needles	9	8.4 (6.5, 10.4)*	0.8 (0.5, 1.0)*	0.6 (0.5, 0.8)*
	Mixed			7	7.7 (5.6, 9.7)*	0.6 (0.4, 0.9)*	0.6 (0.4, 0.7)*
	Bs-stand			7	5.2 (3.4, 6.9)*	0.6 (0.3, 0.8)*	0.6 (0.5, 0.8)*
	Bf-stand	2011	Leaves	9	10.0 (8.0, 12.0)*	1.2 (0.9, 1.4)*	2.5 (1.9, 3.2)*
	Mixed			7	11.7 (9.5, 14.0)*	0.8 (0.5, 1.1)*	2.0 (1.5, 2.4)*
	Bs-stand			7	9.4 (7.6, 11.3)*	0.8 (0.5, 1.0)*	1.5 (1.1, 1.8)*
	Bf-stand	2011	Frass	9	10.7 (8.7, 12.7)*	1.1 (0.8, 1.4)*	0.7 (0.6, 0.9)*
	Mixed			7	9.8 (7.7, 12.0)*	0.9 (0.6, 1.2)*	0.7 (0.5, 0.8)*
	Bs-stand			7	6.8 (5.1, 8.6)*	0.8 (0.5, 1.0)*	0.6 (0.5, 0.7)*
Pairwise comparisons in means	Bf-stand vs mixed	2011	Needles	7	0.8 (-2.1, 3.6)	0.1 (-0.3, 0.5)	0.03 (-0.2, 0.3)
	Bf-stand vs Bs-stand			7	3.3 (0.7, 5.9)*	0.2 (-0.2, 0.6)	-0.01 (-0.2, 0.2)
	Mixed vs Bs-stand			7	2.5 (-0.1, 5.1)	0.1 (-0.3, 0.4)	-0.04 (-0.2, 0.2)
	Bf-stand vs mixed	2011	Leaves	7	-1.8 (-4.8, 1.2)	0.4 (-0.03, 0.7)	0.6 (-0.1, 1.3)
	Bf-stand f vs Bs-stand			7	0.6 (-2.2, 3.3)	0.4 (0.1, 0.8)*	1.1 (0.5, 1.7)*
	Mixed vs Bs-stand			7	2.3 (-0.5, 5.1)	0.1 (-0.3, 0.4)	0.5 (0.1, 0.9)*
	Bf-stand vs mixed	2011	Frass	7	0.9 (-2.0, 3.8)	0.2 (-0.2, 0.6)	0.1 (-0.1, 0.3)
	Bf-stand vs Bs-stand			7	3.9 (1.2, 6.6)*	0.3 (-0.03, 0.7)	0.1 (-0.1, 0.4)
	Mixed vs Bs-stand			7	3.0 (0.4, 5.7)*	0.1 (-0.2, 0.5)	0.1 (-0.2, 0.3)

Supplementary Table 5 Estimated marginal means (EMMs) of nutrient fluxes (N, P and K, in kg ha^{-1}) in litterfall estimated from the linear mixed-effects models (n = 980) and pairwise comparisons among stand types at the beginning of the study period. As litter type and stand type interact with year in all three models, these EMMs and their comparisons are valid for 2011 only. Values in parentheses indicate the 95% confidence interval adjusted for simultaneous inference and stars indicate estimates significantly different from zero at $\alpha = 0.05$.

	**Stand type**	**Year**	**Litter type**	**DF**	**Nitrogen**	**Phosphorus**	**Potassium**
Estimated marginal means (EMMs)	Bf-stand	2011	Needles	9	5.3 (3.6, 7.0)*	0.5 (0.3, 0.7)*	0.4 (0.3, 0.6)*
	Mixed			7	4.6 (2.9, 6.2)*	0.4 (0.2, 0.6)*	0.4 (0.3, 0.5)*
	Bs-stand			7	2.2 (1.0, 3.4)*	0.3 (0.1, 0.4)*	0.3 (0.2, 0.4)*
	Bf-stand	2011	Frass	9	3.9 (2.5, 5.4)*	0.4 (0.2, 0.6)*	0.3 (0.2, 0.4)*
	Mixed			7	2.4 (0.9, 3.8)*	0.3 (0.1, 0.5)*	0.2 (0.1, 0.3)*
	Bs-stand			7	1.8 (0.6, 3.0)*	0.2 (0.1, 0.4)*	0.2 (0.1, 0.3)*
Pairwise comparisons in means	Bf-stand vs mixed	2011	Needles	7	0.8 (-1.6, 3.1)	0.1 (-0.2, 0.4)	0.1 (-0.1, 0.2)
	Bf-stand vs Bs-stand			7	3.1 (1.0, 5.2)*	0.2 (-0.02, 0.5)	0.2 (-0.01, 0.3)
	Mixed vs Bs-stand			7	2.3 (0.4, 4.3)*	0.1 (-0.1, 0.4)	0.1 (-0.1, 0.2)
	Bf-stand vs mixed	2011	Frass	7	1.6 (-0.5, 3.6)	0.1 (-0.1, 0.4)	0.1 (-0.03, 0.3)
	Bf-stand vs Bs-stand			7	2.2 (0.3, 4.0)*	0.2 (-0.04, 0.4)	0.1 (-0.01, 0.3)
	Mixed vs Bs-stand			7	0.6 (-1.2, 2.4)	0.1 (-0.2, 0.3)	0.01 (-0.1, 0.1)

Supplementary Table 6 Slopes of the relationship between nutrient concentration (nitrogen, phosphorus and potassium, in g kg^{-1}) and C:N ratio in living tree needles and outbreak progression (time, in years) for each stand type and species, estimated from the linear mixed-effects models (n = 1693). Pairwise comparisons among stand types and/or between species were performed post hoc when the interaction with time was included in the model. Values in parentheses indicate the 95% confidence interval adjusted for simultaneous inference and stars indicate estimates significantly different from zero at α = 0.05.

	Stand type	Species	n	Nitrogen	Phosphorus	Potassium	C:N ratio
Slope estimates (year)	Bf-stand	*A. balsamea*	312	0.4 (0.3, 0.5)*	0.05 (0.04, 0.06)*	-0.04 (-0.07, -0.01)*	-2.3 (-2.7, -1.9)*
	Mixed		331	0.5 (0.4, 0.6)*			-2.7 (-3.1, -2.3)*
	Bs-stand		105	0.5 (0.4, 0.5)*			-2.9 (-3.5, -2.3)*
	Bf-stand	*P. mariana*	175	0.01 (-0.1, 0.1)	0.009 (0.004, 0.01)*	-0.12 (-0.16, -0.09)*	-0.5 (-1.0, -0.01)*
	Mixed		253	0.1 (0.06, 0.2)*			-0.9 (-1.4, -0.5)*
	Bs-stand		517	0.07 (0.03, 0.1)*			-1.2 (-1.6, -0.7)*
Slope comparisons (year)	All stand types pooled	*A. balsamea* vs *P. mariana*		0.4 (0.3, 0.4)*	0.04 (0.03, 0.05)*	0.08 (0.04, 0.12)*	-1.8 (-2.1, -1.4)*
	Bf-stand vs mixed	Both species pooled		-0.1 (-0.2, -0.04)*			0.4 (-0.1, 0.9)
	Bf-stand vs Bs-stand	Both species pooled		-0.07 (-0.1, 0.01)			0.7 (0.1, 1.2)*
	Mixed vs Bs-stand	Both species pooled		0.05 (-0.02, 0.1)			0.3 (-0.3, 0.8)

Supplementary Table 7 Estimated marginal means of nutrient concentration (N, P and K, in g kg^{-1}) and C:N ratio in tree needles in 2010 estimated from the linear mixed-effects models (n = 1693) and pairwise comparisons among stand types and species at the beginning of the study period. Since there is no interaction between stand type and year in the models for P and K, comparisons remain the same across all years. Values in parentheses indicate the 95% confidence interval adjusted for simultaneous inference and stars indicate estimates significantly different from zero at $\alpha = 0.05$.

	Stand type	Year	Species	DF	Nitrogen	Phosphorus	Potassium	C:N ratio
Estimated marginal means (EMMs)	Bf-stand	2010	*A. balsamea*	9	9.5 (8.5, 10.5)*	1.1 (0.8, 1.4)*	3.6 (3.2, 4.0)*	58.6 (52.9, 64.3)*
	Mixed			7	9.3 (8.2, 10.3)*	0.9 (0.6, 1.2)*	3.4 (3.0, 3.8)*	59.4 (53.4, 65.4)*
	Bs-stand			7	7.9 (6.9, 8.9)*	0.9 (0.6, 1.2)*	3.3 (2.8, 3.7)*	71.8 (65.6, 78.0)*
	Bf-stand	2010	*P. mariana*	9	8.8 (7.8, 9.8)*	1.2 (0.9, 1.5)*	4.6 (4.1, 5.1)*	63.1 (57.1, 69.2)*
	Mixed			7	8.6 (7.5, 9.6)*	0.9 (0.6, 1.2)*	4.4 (3.9, 4.9)*	63.9 (57.6, 70.2)*
	Bs-stand			7	7.2 (6.3, 8.0)*	1.1 (0.9, 1.4)*	4.3 (3.8, 4.7)*	76.3 (70.7, 81.9)*
Pairwise comparisons in means	Bf vs mixed	2010	*A. balsamea*	7	0.2 (-1.1, 1.5)	0.2 (-0.1, 0.6)	0.2 (-0.3, 0.7)	-0.8 (-8.2, 6.7)
	Bf vs Bs			7	1.6 (0.4, 2.9)*	0.2 (-0.1, 0.6)	0.3 (-0.1, 0.8)	-13.2 (-20.7, -5.8)*
	Mixed vs Bs			7	1.4 (0.2, 2.6)*	-0.02 (-0.4, 0.3)	0.1 (-0.3, 0.6)	-12.4 (-19.8, -5.1)*
	Bf vs mixed	2010	*P. mariana*	7	Same as *A. balsamea*	0.3 (-0.1, 0.7)	Same as *A. balsamea*	Same as *A. balsamea*
	Bf vs Bs			7		0.1 (-0.3, 0.5)		
	Mixed vs Bs			7		-0.2 (-0.5, 0.2)		
	Bf-stand	2010	*A. balsamea* vs *P. mariana*	347	0.73 (0.43, 1.03)*	-0.1 (-0.2, -0.03)*	-1.0 (-1.2, -0.7)*	-4.5 (-6.5, -2.5)*
	Mixed			347		-0.07 (-0.1, -0.01)*		
	Bs-stand			347		-0.2 (-0.3, -0.2)*		

Supplementary Table 8 Estimated marginal means (EMMs) and slopes of the relationship between summer soil temperature (°C) and local cumulative defoliation (%) for each stand type and month, estimated from the linear mixed-effects model (n = 3459). Pairwise comparisons among stand types were performed post hoc. Values in parentheses indicate the 95% confidence interval adjusted for simultaneous inference and stars indicate estimates significantly different from zero at α = 0.05. As the model includes a three-way interaction between defoliation, stand type and month, defoliation was set at 0% to calculate EMMs and their comparisons.

Stand type	Month	n	Slopes	EMMs
Bf-stand	June	339	0.05 (0.04, 0.06)*	4.5 (3.3, 5.7)*
Mixed		355	0.04 (0.03, 0.05)*	4.6 (3.3, 5.8)*
Bs-stand		475	0.05 (0.03, 0.06)*	5.9 (4.9, 6.9)*
Bf-stand	July	331	0.03 (0.02, 0.04)*	9.7 (8.7, 10.8)*
Mixed		337	0.03 (0.02, 0.03)*	9.4 (8.3, 10.5)*
Bs-stand		463	0.02 (0.02, 0.03)*	10.1 (9.2, 11.1)*
Bf-stand	August	337	0.03 (0.02, 0.03)*	10.8 (9.8, 11.8)*
Mixed		346	0.03 (0.03, 0.04)*	10.2 (9.1, 11.3)*
Bs-stand		476	0.02 (0.01, 0.03)*	11.2 (10.2, 12.1)*
Bf-stand vs mixed	June		0.01 (-0.002, 0.02)	-0.1 (-1.6, 1.4)
Bf-stand vs Bs-stand			0.003 (-0.01, 0.02)	-1.4 (-2.8, -0.02)*
Mixed vs Bs-stand			-0.007 (-0.02, 0.006)	-1.3 (-2.7, 0.02)
Bf-stand vs mixed	July		0.001 (-0.007, 0.009)	0.4 (-1.0, 1.7)
B-stand f vs Bs-stand			0.004 (-0.004, 0.01)	-0.4 (-1.6, 0.9)
Mixed vs Bs-stand			0.003 (-0.005, 0.01)	-0.7 (-2.0, 0.5)
Bf-stand vs mixed	August		-0.003 (-0.009, 0.003)	0.6 (-0.7, 1.9)
Bf-stand vs Bs-stand			0.009 (0.002, 0.02)*	-0.4 (-1.6, 0.8)
Mixed vs Bs-stand			0.01 (0.005, 0.02)*	-1.0 (-2.1, 0.2)
