# Supplementary material for: Defoliation-induced changes in foliage quality may trigger broad-scale insect outbreaks
Source: Commun Biol. 2022 May 16;5:463. doi: 10.1038/s42003-022-03407-8 (PMC9110339; doi:10.1038/s42003-022-03407-8)
Supplement: Supplementary file 3 — Description of Additional Supplementary Files [file 42003_2022_3407_MOESM3_ESM.pdf]

Description of Additional Supplementary Files

File name: Supplementary Data 1
Description: Data input for figures.
