# Supplementary material for: Defoliation-induced changes in foliage quality may trigger broad-scale insect outbreaks
Source: Commun Biol. 2022 May 16;5:463. doi: 10.1038/s42003-022-03407-8 (PMC9110339; doi:10.1038/s42003-022-03407-8)
Supplement: Supplementary file 5 — Reporting Summary [file 42003_2022_3407_MOESM5_ESM.pdf]

nature portfolio

Corresponding author(s): Louis De Grandpré
Last updated by author(s): Mar 23, 2022

Reporting Summary

Nature Portfolio wishes to improve the reproducibility of the work that we publish. This form provides structure for consistency and transparency in reporting. For further information on Nature Portfolio policies, see our Editorial Policies and the Editorial Policy Checklist.

Statistics

For all statistical analyses, confirm that the following items are present in the figure legend, table legend, main text, or Methods section.

n/a	Confirmed	
☐	☒	The exact sample size (n) for each experimental group/condition, given as a discrete number and unit of measurement
☐	☒	A statement on whether measurements were taken from distinct samples or whether the same sample was measured repeatedly
☐	☒	The statistical test(s) used AND whether they are one- or two-sided<br>*Only common tests should be described solely by name; describe more complex techniques in the Methods section.*
☐	☒	A description of all covariates tested
☐	☒	A description of any assumptions or corrections, such as tests of normality and adjustment for multiple comparisons
☐	☒	A full description of the statistical parameters including central tendency (e.g. means) or other basic estimates (e.g. regression coefficient) AND variation (e.g. standard deviation) or associated estimates of uncertainty (e.g. confidence intervals)
☐	☒	For null hypothesis testing, the test statistic (e.g. F, t, r) with confidence intervals, effect sizes, degrees of freedom and P value noted<br>*Give P values as exact values whenever suitable.*
☒	☐	For Bayesian analysis, information on the choice of priors and Markov chain Monte Carlo settings
☐	☒	For hierarchical and complex designs, identification of the appropriate level for tests and full reporting of outcomes
☒	☐	Estimates of effect sizes (e.g. Cohen's d, Pearson's r), indicating how they were calculated

Our web collection on statistics for biologists contains articles on many of the points above.

Software and code

Policy information about availability of computer code

Data collection	n/a
Data analysis	Rsoftware ver4.1.2

For manuscripts utilizing custom algorithms or software that are central to the research but not yet described in published literature, software must be made available to editors and reviewers. We strongly encourage code deposition in a community repository (e.g. GitHub). See the Nature Portfolio guidelines for submitting code & software for further information.

Data

Policy information about availability of data

All manuscripts must include a data availability statement. This statement should provide the following information, where applicable:

- Accession codes, unique identifiers, or web links for publicly available datasets
- A description of any restrictions on data availability
- For clinical datasets or third party data, please ensure that the statement adheres to our policy

Data will be available on the open government of Canada portal.

Field-specific reporting

Please select the one below that is the best fit for your research. If you are not sure, read the appropriate sections before making your selection.

☐ Life sciences ☐ Behavioural & social sciences ☒ Ecological, evolutionary & environmental sciences

For a reference copy of the document with all sections, see nature.com/documents/nr-reporting-summary-flat.pdf

Ecological, evolutionary & environmental sciences study design

All studies must disclose on these points even when the disclosure is negative.

Study description

We tested the prediction that herbivory causes a positive feedback on outbreak severity as nutrient concentration in plant tissues increases through improved soil nutrient availability from frass and litter deposition. Over seven years of a spruce budworm outbreak, we quantified litter nutrient fluxes, soil nitrogen availability and host tree foliar nutrient status along a forest susceptibility gradient.

We used linear mixed-effects models to explore the relationships between nutrients in litterfall and outbreak progression (time) and to test whether this relationship differed with stand composition and among litter types. Models were fitted separately for each nutrient (N, P and K) with either concentration or quantity of nutrients as the response variable. Litter type (needles, leaves and frass), stand composition (fir-dominated, mixed or spruce-dominated), year, their three-way interaction and all possible two-way interactions were considered as fixed effects. Sampling points nested in sites were included as random intercepts to account for variation in nutrient concentration or quantity among sampling points and sites and repeated measurements at each sampling point.

A similar modeling structure was used to assess the effect of outbreak progression on tree foliage nutrients. Response variables were N, P and K concentrations and C:N ratio in live needles. Stand composition, tree species, year and their interactions were evaluated as possible fixed effects while sampled trees nested within a site were included as random intercepts to account for variation in needle nutrient concentration among sites and trees as well as repeated measurements on the same trees.

A linear mixed-effect model was also used to test for changes in summer soil temperature with increasing tree defoliation. Average annual percent defoliation (median of defoliation classes) in a 5-meter radius around each sample point in interaction with month (June, July and August) and stand composition were used as fixed effects and temperature probes nested in sampling points and site as random effects.

A generalized linear mixed model (GLMM) with a gamma distribution and log link function was fitted with Soil Inorganic Nitrogen as the response variable, cumulative defoliation, percentage of fir and basal area in a 5-meter radius around each sample point as fixed effects. Sampling points nested in sites were added as random effects.

Research sample

10 permanent sample plots (1ha-4000m2) established in spruce budworm affected forest stands (Abies balsamea-dominated, Picea mariana-dominated, and mixed) originating from fire and timber harvest, between 80-200+ years of age. In each plot, 21 litter traps were installed and contents were collected yearly. Sampling strategy ensured adequate coverage of forest composition types, stand age and vulnerability to spruce budworm damage. Research sample is meant to represent boreal forest stands typically defoliated by SBW.

Sampling strategy

Litter traps were distributed along a linear transect of 70m within each forest stand. Litter was collected yearly, dried, sorted, weighed, and analyzed for nutrient contents. Soil temperature was measured using underground sensors located next to each trap. In addition, ion-exchange resin beads were also located next to traps and analyzed yearly. Tree mortality and defoliation was evaluated yearly for all trees in the sampling plot. Branches were collected on specific sampling trees located along two parallel transects. Fine-scale defoliation was evaluated using these branches and their needles analyzed for nutrient content. Sample size was determined using data from the literature. Trap layout (7 clusters of 3 traps each separated by 10m) ensured that we captured the small to large-scale variability in forest and soil conditions.

Data collection

Sampling occured yearly between the months of May and September. Data collection was performed by a team of field technicians, research assistants and research professionals. Field data was entered into a relational database with extensive validation.

Timing and spatial scale

From 2011 to 2017, traps were maintained all year and their content collected every year in early September, approximately 4 to 6 weeks after the end of defoliation. Resin bags were also collected at the same time, allowing us to associate the ion-exchange data to the litter-nutrient data. Defoliation must be complete before these analyses can be performed. Permanent sampling plots are approximately 30 km apart and are located at the epicenter of the SBW outbreak. We cover variability from the regional (80000ha) to the local scale (0.5ha).

Data exclusions

No data was excluded.

Reproducibility

It was not possible for us to reproduce this long-term in situ experiment as SBW outbreaks have a periodicity of 30-40 years. Despite this, the methods used are easily reproducible.

Randomization	Sampling plots were established based on a gradient of forest composition taking into account stand age and origin. The exact location of litter traps were determined following a stratified, randomized design.
Blinding	Blinding is not applicable in this study as no treatment assignment was necessary.

Did the study involve field work? ☒ Yes ☐ No

Field work, collection and transport

Field conditions	Field work was largely unaffected by meteorological conditions. Measures were taken to avoid contamination of samples (dried promptly in case of rainfall, etc.).
Location	P001 49.6, -67.9<br>P013 49.5, -67.7<br>P015 49.7, -68.1<br>P002 49.5, -67.8<br>P022 49.7, -68.1<br>P003 49.6, -67.8<br>P031 49.4, -67.9<br>P006 49.5, -67.8<br>P092 49.4, -67.6<br>P095 49.4, -67.9
Access & import/export	Permanent sampling plots are located on public land and protected from timber harvest under a conservation agreement with the provincial government and industry.
Disturbance	One branch per year was collected on each sample tree.

Reporting for specific materials, systems and methods

We require information from authors about some types of materials, experimental systems and methods used in many studies. Here, indicate whether each material, system or method listed is relevant to your study. If you are not sure if a list item applies to your research, read the appropriate section before selecting a response.

Materials & experimental systems

n/a	Involved in the study	
☒	☐	Antibodies
☒	☐	Eukaryotic cell lines
☒	☐	Palaeontology and archaeology
☒	☐	Animals and other organisms
☒	☐	Human research participants
☒	☐	Clinical data
☒	☐	Dual use research of concern

Methods

n/a	Involved in the study	
☒	☐	ChIP-seq
☒	☐	Flow cytometry
☒	☐	MRI-based neuroimaging
